# A Case Report of a Lebanon Viper (*Montivipera bornmuelleri*) Envenomation in a Child

**DOI:** 10.5811/cpcem.2022.2.56176

**Published:** 2022-08-06

**Authors:** Faysal Tabbara, Sarah S Abdul Nabi, Riad Sadek, Ziad Kazzi, Tharwat El Zahran

**Affiliations:** * American University of Beirut, Department of Emergency Medicine, Beirut, Lebanon; † Emory University, Department of Emergency Medicine, Atlanta, Georgia; ‡ American University of Beirut, Department of Biology, Beirut, Lebanon

**Keywords:** Lebanon viper, Montivipera bornmuelleri, envenomation, case report

## Abstract

**Introduction:**

Snake envenomation is a serious public health concern. In the Middle East little is known about snakebite envenomation, which raises several challenges for emergency physicians caring for these patients.

**Case report:**

We report the case of a five-year-old boy bitten by a rare snake, *Montivipera bornmuelleri*, who presented to an emergency department in Lebanon. We also discuss the proper management of snake envenomation.

**Conclusion:**

This case is unique as snakebites in Lebanon are poorly studied, and little is known about the epidemiology and clinical manifestations of local snakebites.

## INTRODUCTION

In 2017, snake envenomation was listed by the World Health Organization (WHO) as a neglected tropical disease.^1^ Globally, snake envenomation represents a serious public health concern with approximately five million snakebites occurring every year. An estimate of 81,000 to 138,000 people die yearly because of snakebites, and approximately three times as many amputations and other disabilities are caused by snakebites annually.^2^,^3^ Unfortunately, in the Middle East there is insufficient data describing snakebite envenomation, which constitutes a challenge for clinicians who care for these patients in the emergency department (ED). Indeed, the lack of knowledge about these snakes and the lack of timely appropriate antivenom at the Lebanese hospitals are two of the main causes of morbidity. One of the few studies available has described the types of snakes in Lebanon, incidence, clinical outcomes, and available antivenoms.^4^ We report the case of a five-year-old child who was bitten by a rarely encountered Lebanese snake, *Montivipera bornmuelleri*, which is also known as the Lebanon viper.

## CASE REPORT

**Image 1:**
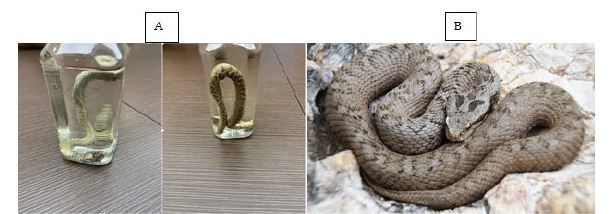
A Lebanon viper (*Montivipera bornmuelleri*) snake killed at 2000-meter altitude in northern Lebanon (A) and an image of the *Montivipera bornmuelleri* taken in nature (B).

**Image 2:**
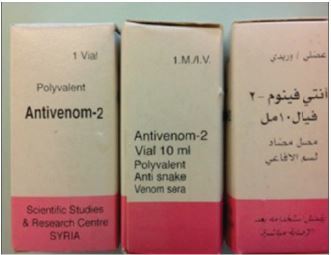
Images of the polyvalent anti-venom (Antivenom-2) manufactured by the Scientific Studies and Research Center in Damascus, Syria, which was obtained by the child’s parents from the Lebanese Ministry of Public Health.

A five-year-old boy presented to the ED 13 hours after sustaining a snakebite to his right middle finger. The incident occurred in the Cedars (Arez) area (northern Lebanon) at around 2000 meters above sea level. Prior to arrival to our institution, the patient was taken to another hospital where he was treated with dexamethasone and promethazine. The parents killed the snake and stored it in a bottle filled with an isopropyl alcohol-based solution (Image 1). Beside it is a photograph of *Montivipera bornmuelleri* taken in nature (Image 1). The parents obtained the polyvalent anti-venom Antivenom-2 from the Lebanese Ministry of Public Health. Antivenom-2 is manufactured in Syria [Scientific Studies and Research Center, Damascus]) (Image 2).

**Image 3:**
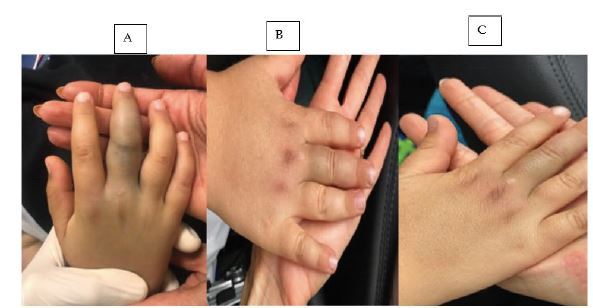
A five-year-old patient with a *Montivipera bornmuelleri* envenomation resulting in swelling over his right middle phalanx (A) with follow-up after anti-venom administration the next day (B) and three days later (C).

Upon arrival to the ED, the patient was complaining of mild pain in his right middle finger. He denied vomiting, abdominal pain, shortness of breath, loss of consciousness, headache, or other complaints. The patient was hemodynamically stable with a blood pressure of 105/63 millimeters of mercury, temperature 36.5°C, heart rate 88 beats per minute, and oxygen saturation 99% on room air. Pertinent physical examination findings included a well-appearing and comfortable child with a normal rate and rhythm and normal pulses. He had good bilateral air entry on lung auscultation, and his neurologic exam was unremarkable. On musculoskeletal exam, his right middle finger was swollen with ecchymosis of the middle phalanx (Image 3). The finger had intact range of motion over the proximal and distal interphalangeal joints, with intact sensation. There was no erythema or warmth, and the capillary refill was less than two seconds.

Initial laboratory studies revealed a complete blood count (CBC) with a white blood cell count (WBC) of 9.7 thousand cells per cubic millimeters. (K/mm3) (reference range 5 to 17 K/mm3), hemoglobin (Hb) 13.9 grams per deciliter (g/dL) (10–15 g/dL), platelets (plt) 346,000 /μL (150 to 400,000/μL), creatinine 0.4 milligrams/dL (mg/dL) (0.6 to 1.2 mg/dL), D-dimer 210 nanograms per milliliter (ng/mL) (normal less than 255 ng/mL), prothrombin time 11.9 seconds (control 11.4 seconds), activated partial thromboplastin time 32.1 seconds (control 32.5 seconds), international normalized ratio (INR) 1.0 (0.9 to 1.2), fibrinogen 2.38 grams per liter (g/L) (1.7 to 4 g/L) and a creatinine phosphokinase (CPK) 712 international units per kilogram (IU/kg) (20 to 205 IU/kg).

The snake was identified by the consulting medical toxicology team as *Montivipera bornmuelleri*. A digital plethysmography was done by the vascular surgery consultant, which showed normal digital pressures in both hands as well as patent radial and ulnar arteries with normal triphasic flow pattern. Due to the swelling and the concern for additional progression, the child was given 5 mL of Antivenom-2.

**CPC-EM Capsule**

What do we already know about this clinical entity?

Montivipera bornmuelleri *snake is rare and was shown in experimental studies to exhibit hemodynamic, hemolytic, pro-inflammatory and neurotoxic properties*.

What makes this presentation of disease reportable?

*We detail the clinical presentation, management and the use of the polyvalent anti-venom specific for rare snakes inhabiting the Middle East area*.

What is the major learning point?

*The major learning point from this case report is to highlight the clinical manifestation and management of this rare envenomation*.

How might this improve emergency medicine practice?

*It would benefit the emergency physicians as a first guide to the presentation and management of this rare case of snake envenomation*.

Repeat laboratory studies taken eight hours later (20 hours after the bite) revealed a WBC of 15,400 K/mcL. Hb of 12.5 g/dL, plt 340,000/μL, D-dimer 182 ng/mL, INR 1.0, fibrinogen 2.05 g/L, and CPK 849 IU/L. No systemic toxicity developed. The swelling did not extend beyond the middle phalanx, and ecchymosis decreased. Repeated laboratory studies including coagulation profile remained normal. The increase in WBC was attributed either to being stress-induced or due to the corticosteroids taken at the outside hospital. The patient was successfully discharged home from the ED and instructed to obtain repeat CBC, coagulation profile, and CPK the next day and to follow up with the toxicology team. Follow-up images of the right middle phalanx were obtained the next day (Image 3b) and three days after discharging the patient (Image 3c).

## DISCUSSION

Snakebites in Lebanon have been poorly studied, and little is known about the epidemiology or clinical manifestations of local snakebites.^5^ Lebanon has around 25 species of snakes, three of which are venomous vipers: *Daboia palaestinae*, *Macrovipera lebetina*, and *M. bornmuelleri*. One study^4^ found that most cases of envenomation were attributed to *M. lebetina* and *D. palaestinae*. The clinical manifestations of these snakebites include tachycardia (33.3%), hypotension (20.8%), anaphylaxis (12.5%), headache (4.2%), and nausea, vomiting, and abdominal pain (4.2% each). Hematological abnormalities include leukocytosis (37.5%), and coagulopathy and thrombocytopenia (12.5% each). Among the reported complications are dizziness or impaired consciousness, compartment syndrome, deep venous thrombosis, acute respiratory distress syndrome, sepsis, cellulitis, upper gastrointestinal bleeding, vaginal bleeding, and congestive heart failure.

Although little is known about the biology of this rare species, it is likely to share aspects with other members of the Viperidae family and the subfamily Viperinae. This species is endemic to high altitudes (above 1800 meters) and is mainly found in Lebanon, specifically in Mount Lebanon, and less abundantly in Palestine. Members of the *Montivipera* genus usually have a short tail and can grow to a maximum of 75 centimeters in length.^6^

*M. bornmuelleri* viper venom is known to reduce blood pressure by displaying vasorelaxant effects that act synergistically on different pathways. It can act on endothelial cells and induce the release of the vasoactive mediator, nitric oxide, reduce calcium ion influx through voltage-dependent calcium channels, and inhibit contraction induced by angiotensin I.^7^ The venom has been shown to exhibit strong antibacterial, hemolytic, anticoagulant, and pro-inflammatory activities.^8^ Recent in vivo studies have explored some potential anti-cancer and immunomodulator properties that the *M. bornmuelleri* viper venom might exhibit.^8^,^9^ It is well known that viper venom can act on the nervous system.^7^,^8^,^9^ However, no studies exist outlining the effect of *M. bornmuelleri* venom on the nervous system. Some researchers suggest that this neurologic effect might be attributed to the effect that this venom has on calcium/potassium and sodium channel blockage.^7^ Future studies are needed to highlight other potential biological and clinical manifestations of this rare viper’s envenomation.

To our knowledge, few or no reported cases exist in the literature describing the clinical manifestations or mortality caused by envenomation by the *M. bornmuelleri* as it is considered one of the rarest snakes inhabiting high altitudes in the Middle East.^10^ In fact, Abi-Rizk et al, in a study performed on mice, reported that the median lethal dose (LD50) of *M. bornmuelleri* venom injected intramuscularly LD50 is 5.93 mg/kg and intraperitoneally (IP) LD50 is around 1.93. The lethality of the *M. bornmuelleri* venom is similar to that of *D. palestinae* with an experimental IP LD50 of 1.9 mg/kg.^9^

Luckily our patient developed only minor local signs and symptoms without any systemic toxicity. The role of the antivenom that was administered by the treating physician is unknown. This patient received the antivenom in this case because it is not a common envenomation; however, it is one of the toxic envenomations that can lead to coagulopathy and death. Furthermore, it is the first snakebite treated at our institution. Antivenom-2 is manufactured from the serum of horses that were immunized with venom from the snakes *D. palaestinae, Macrovipera lebetinus, Vipera xanthina, Vipera amodytes*, and *Cerastes cerastes*. This antivenom had formerly been adopted by the Lebanese Ministry of Public Health and distributed free of charge to hospitals. Due to the war in Syria and the resulting difficulties in procuring this antivenom, it was recently substituted by the Menaven/Biosnake antivenom manufactured in India by VINS Bioproducts Ltd, Hyderabad.^11^

While this antivenom is listed to be active specifically against some of the Lebanese snakes, *M. lebitinus* and *D. palaestinae*, it is not listed for use in cases of bites from the Lebanon viper. In our clinical experience in Lebanon, other products are occasionally used that are not listed to be active specifically against any of the snakes indigenous to Lebanon. A geographically specific and comprehensive database of snakes and antivenoms is urgently needed to address these important gaps in the appropriate management of snakebites, which the WHO has deemed a neglected tropical disease with an asymmetrical impact on developing countries. Of note, the dosing of the antivenom is not dependent upon age or weight but it does vary based on the severity of the envenomation.^11^ For more information about the antivenoms available, we are attaching below in Appendix A the leaflet of the Syrian antivenom along with its translation and in Appendix B the leaflet of the Indian antivenom.

## CONCLUSION

The scenario we report describes an interesting case of a five-year-old male patient who presented after a snakebite envenomation on his right finger, secondary to the *M. bornmuelleri*. He was successfully treated with polyvalent antivenom, after which the swelling did not extend beyond the middle phalanx and the ecchymosis decreased. The patient was discharged home after observation at our ED. This case is unique as snakebites in Lebanon are poorly studied, and little is known about the epidemiology and clinical manifestations of local snakebites, in particular those caused by envenomation by the *M. bornmuelleri*, given that it is considered one of the rarest snakes inhabiting high altitudes in the Middle East.
